# Ferroptosis, Necroptosis, and Pyroptosis in Gastrointestinal Cancers: The Chief Culprits of Tumor Progression and Drug Resistance

**DOI:** 10.1002/advs.202300824

**Published:** 2023-07-12

**Authors:** Xudong Zhu, Shenglong Li

**Affiliations:** ^1^ Department of General Surgery Cancer Hospital of Dalian University of Technology Cancer Hospital of China Medical University Liaoning Cancer Hospital and Institute Shenyang Liaoning Province 110042 China; ^2^ Second Ward of Bone and Soft Tissue Tumor Surgery Cancer Hospital of Dalian University of Technology Cancer Hospital of China Medical University Liaoning Cancer Hospital and Institute Shenyang Liaoning Province 110042 China; ^3^ The Liaoning Provincial Key Laboratory of Interdisciplinary Research on Gastrointestinal Tumor Combining Medicine with Engineering Shenyang Liaoning Province 110042 China

**Keywords:** drug resistance, ferroptosis, gastrointestinal cancers, necroptosis, pyroptosis, targeted therapy

## Abstract

In recent years, the incidence of gastrointestinal cancers is increasing, particularly in the younger population. Effective treatment is crucial for improving patients’ survival outcomes. Programmed cell death, regulated by various genes, plays a fundamental role in the growth and development of organisms. It is also critical for maintaining tissue and organ homeostasis and takes part in multiple pathological processes. In addition to apoptosis, there are other types of programmed cell death, such as ferroptosis, necroptosis, and pyroptosis, which can induce severe inflammatory responses. Notably, besides apoptosis, ferroptosis, necroptosis, and pyroptosis also contribute to the occurrence and development of gastrointestinal cancers. This review aims to provide a comprehensive summary on the biological roles and molecular mechanisms of ferroptosis, necroptosis, and pyroptosis, as well as their regulators in gastrointestinal cancers and hope to open up new paths for tumor targeted therapy in the near future.

## Graphical abstract

Programmed cell death mainly include ferroptosis (A), necroptosis (B), and pyroptosis (C), which are morphologically similar and can induce severe inflammatory responses. Notably, ferroptosis, necroptosis, and pyroptosis also participate in the occurrence and development of cancers. Here, the molecular mechanisms of ferroptosis, necroptosis, and pyroptosis are summarized.

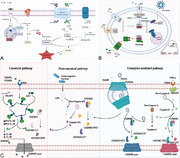

## 1 Introduction

**Figure 1:**
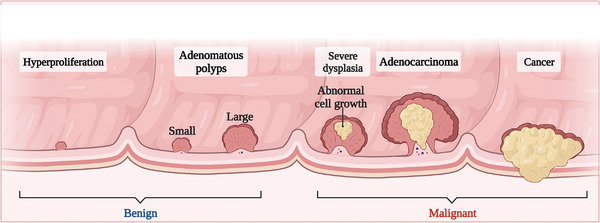
The process colorectal cancer development. The process of colorectal cancer development can be classified as hyperproliferation, adenomatous polyps, severe dysplasia, adenocarcinoma, and cancer.

**Figure 2:**
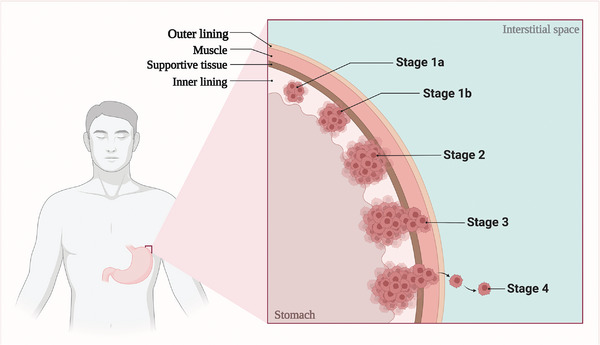
Clinical staging of gastric cancer. According to the degree of invasion, gastric cancer can be classified as stage 1a, stage 1b, stage 2, stage 3, and stage 4.

Gastrointestinal (GI) cancers are malignant neoplasms that arise in the GI tract and digestive organs.^[^ ^1^ ^]^ Several types of GI cancers have a common endodermal origin.^[^ ^2^ ^]^ The most prevalent GI cancers include liver cancer, colorectal cancer (CRC), gastric cancer (GC), and esophageal cancer.^[^ ^3^ ^]^ CRC may arise as a complication of chronic inflammatory bowel diseases, such as ulcerative colitis.^[^ ^4^, ^5^ ^]^ A schematic of the tumorigenic process in CRC is presented in **Figure** **1**. Infection with viruses such as Epstein–Barr virus or Helicobacter pylori is commonly associated with GC.^[^ ^6^, ^7^, ^8^ ^]^ The development process and clinical staging of GC are shown in **Figure** **2**. Current interventions for GI cancers encompass preoperative neoadjuvant therapy, surgical treatment, postoperative adjuvant therapy, targeted medication against epidermal growth factor receptor (EGFR), immunotherapy, and palliative care. Surgical treatment offers the greatest effectiveness for patients with GI cancers.^[^ ^9^, ^10^ ^]^ However, most patients with GI cancers exhibit inconspicuous early symptoms and the majority have already developed local infiltration or distant metastasis by the time of the initial consultation, thereby precluding curative surgery.^[^ ^11^, ^12^ ^]^ Hence, scientists are actively exploring molecular markers for early detection and alternative therapeutic targets for GI cancers.

**Figure 3:**
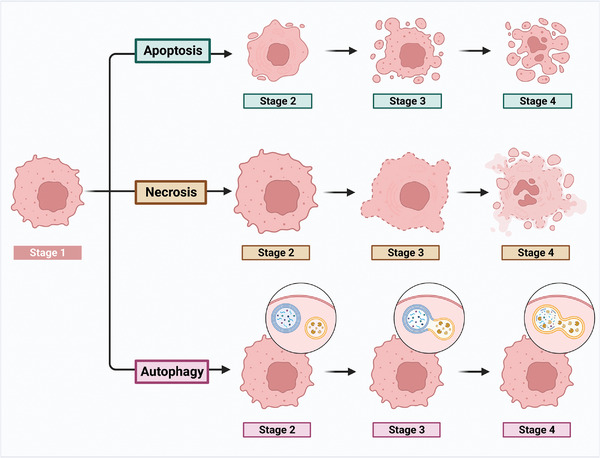
The basic processes of apoptosis, necrosis, and autophagy. The process of tumor cell death can be divided into Stage 1 (cancer cell in basal state) and various stages of apoptosis, necrosis, and autophagy. The three stages of apoptosis are Stage 2 (cell shrinkage and chromatin condensation), Stage 3 (membrane blebbing and nuclear fragmentation), and Stage 4 (apoptotic body formation). The three periods of necrosis are Stage 2 (swelling of the cell and organelles), Stage 3 (rupture of cell membrane), and Stage 4 (cell content release). The three periods of autophagy are Stage 2 (the shape of autophagosome and lysosome), Stage 3 (autophagosome and lysosome fusion), and Stage 4 (autophagy of cell debris/organelles).

Classically, cell death was categorized as apoptosis, also called programmed cell death (PCD), necrosis, or autophagy (**Figure** **3**). Apoptosis is a process of active cell death that can be blocked by cell signaling inhibitors, whereas necrosis is a passive process that is not susceptible to blockade by cell signaling inhibitors.^[^ ^13^, ^14^ ^]^ PCD is a self‐directed and orderly process that is crucial for the development and maintenance of organisms, involving the activation, expression, and regulation of various genes through distinct pathways.^[^ ^15^, ^16^ ^]^ Recently, new molecular mechanisms of cell death have been described. Programmed cell death can be subcategorized into major groups, such as apoptosis, autophagy, necroptosis, pyroptosis, ferroptosis, and others.^[^ ^17^ ^]^ In this review, we focus on ferroptosis, necroptosis, and pyroptosis, and evaluate the regulatory factors that govern these processes in GI cancers, as well as their clinical relevance regarding prognosis and treatment resistance.

## 2 Overview of Ferroptosis

**Figure 4:**
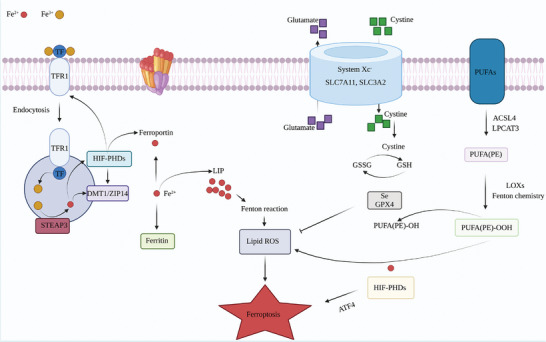
Overview of the molecular mechanisms of ferroptosis. Recent research on ferroptosis has identified several regulatory mechanisms involving GPX4, TFR1, and System Xc‐. The inactivation of GPX4 or inhibition of System Xc‐ can result in ferroptosis due to the accumulation of lipid peroxides, while TFR1 plays a role in regulating properties related to ferroportin. All these key regulators together construct a network of ferroptosis in GI cancers.

Ferroptosis is a form of regulated cell death that is characterized by the dysregulation of iron and lipid metabolism.^[^ ^18^, ^19^ ^]^ Iron is an essential trace element and plays a crucial role in various physiological processes. Under certain conditions, such as excess iron or oxidative stress, iron promotes the formation of reactive oxygen species (ROS) through the Fenton reaction.^[^ ^20^ ^]^ ROS can lead to the peroxidation of lipids by activating lipoxygenase (LOX) and generating lipid peroxides. Polyunsaturated fatty acids (PUFAs) and cholesterol in the cytoplasm and membrane of organelles, especially mitochondria, are particularly susceptible to lipid peroxidation. The accumulation of lipid peroxides leads to membrane damage and the disruption of cell and mitochondrial membrane integrity, ultimately resulting in ferroptosis.^[^ ^21^, ^22^, ^23^, ^24^ ^]^ Unlike other forms of programmed cell death, such as apoptosis and autophagy, ferroptosis has unique morphological, biochemical, and genetic features. It does not exhibit the typical features of apoptosis, such as chromatin condensation, cytochrome C cleavage, or caspase‐3 activation. Instead, ferroptosis involves the specific and selective degradation of lipids, which leads to the loss of selective permeability and mitochondrial dysfunction. The reduction or loss of mitochondrial cristae, rupture of the outer mitochondrial membrane, and condensation of the mitochondrial membrane are all characteristic of ferroptosis. Recent research on ferroptosis has identified several regulatory mechanisms involving GPX4,^[^ ^17^, ^25^ ^]^ transferrin receptor 1 (TFR1),^[^ ^26^ ^]^ and System Xc‐. The inactivation of GPX4 or inhibition of System Xc‐ can result in ferroptosis due to the accumulation of lipid peroxides,^[^ ^27^, ^28^ ^]^ while TFR1 plays a role in regulating properties related to ferroportin^[^ ^29^, ^30^ ^]^ (**Figure** **4**). In summary, ferroptosis is a complex and multifactorial process that involves the dysregulation of iron and lipid metabolism, oxidative stress, and mitochondrial dysfunction. The accumulation of lipid peroxides leads to the degradation of cell and mitochondrial membrane integrity and ultimately results in ferroptosis.

## 3 Overview of Necroptosis

**Figure 5:**
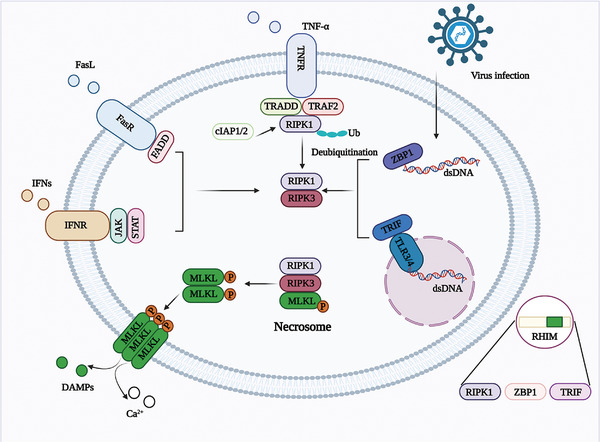
Overview of the molecular mechanisms of necroptosis. Necroptosis is triggered by the binding of cell surface death receptors (including FasR, TNFR, IFN receptor, and TLR) and ZBP1 and RHIM‐containing downstream proteins to RIPK3. Subsequently, necrosome formation and cell lysis result.

Necroptosis is a programmed cell death process that involves the activation of mixed lineage kinase domain‐like protein (MLKL)/pMLKL by receptor‐interacting protein kinase 1 (RIPK1)/receptor‐interacting protein kinase 3 (RIPK3)‐mediated phosphorylation signaling pathway.^[^ ^31^, ^32^ ^]^ The cell death pattern of necroptosis is characterized by lysosomal membrane decline, cytoplasmic vacuolization, plasma membrane disassembly, and cellular rupture, and is driven by the kinase activity of RIPK1. TNF‐α binding to TNFR1 recruits downstream protein molecules to form complex I, including TRADD, RIPK1, cellular inhibitor of apoptosis proteins, TRAF2/5, and LU‐BAC proteins.^[^ ^17^, ^33^ ^]^ When the activity of caspase‐8 is suppressed or inhibited, RIPK1 recruits RIPK3, and the resulting complex recruits MLKL via the RIP homotypic interaction motif (RHIM) in a process that leads to the formation of complex IIb, which is also called the necrosome.^[^ ^34^, ^35^ ^]^ MLKL undergoes activation via phosphorylation at T357 and S358 sites, leading to its oligomerization and localization to the membrane. Oligomerized MLKL specifically binds to lipids, and by forming pores, it disrupts the integrity of the cell membrane, which finally induces necroptosis.^[^ ^36^, ^37^, ^38^ ^]^ RIPK1 lacks kinase activity in complex I. When a necroptosis inhibitor (necrostatin‐1) suppresses the activity of RIPK1, the TNF‐induced NF‐κB signaling pathway remains unaffected. However, the formation of complex IIb can be blocked by inhibiting necroptosis. **Figure** **5** provides an overview of the molecular mechanisms of necroptosis.

## 4 Overview of Pyroptosis

Pyroptosis is a form of programmed cell death (PCD) mediated by caspase‐1 that is characterized by rapid plasma membrane rupture, followed by the release of cellular contents and proinflammatory cytokines. This, in turn, triggers an inflammatory cascade culminating in cellular destruction.^[^ ^39^, ^40^ ^]^ Pyroptosis pathways include both the classical pathway, which is mediated by caspase‐1, and the nonclassical pathway, which involves caspase‐4, 5, and 11.^[^ ^41^, ^42^ ^]^ The classical pathway of pyroptosis is initiated by the assembly of an inflammasome, which activates caspase‐1. In contrast, in the nonclassical pathway, LPS leads to caspase‐4, 5, and 11 activation, which cleave GSDMD into N‐terminal and C‐terminal ends, inducing pyroptosis.^[^ ^43^, ^44^, ^45^, ^46^, ^47^ ^]^ Inflammasomes comprise PRRs such as NLRP1 and NLRP3, ASC, and pro‐caspase‐1. The PRRs recognize various intracellular signals and bind to ASC through its N‐terminal PYD. ASC recruits pro‐caspase‐1 through the interaction of the CARD structural domains, leading to the activation of caspase‐1. Caspase‐1 cleaves GSDMD, and GSDMD‐N binds to cell membrane phospholipids to form pores, disrupting cell integrity and initiating pyroptosis. Additionally, caspase‐1 promotes the secretion of interleukins, leading to an inflammatory response.^[^ ^48^, ^49^, ^50^, ^51^, ^52^, ^53^ ^]^

**Figure 6:**
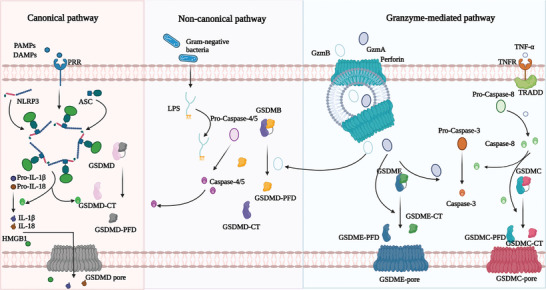
Overview of the molecular mechanisms of pyroptosis. The main pathway involved in pyroptosis. Canonical pathway: Interaction of pathogen‐associated molecular pattern (PAMP) and damage‐associated molecular pattern (DAMP) with pattern recognition receptors on the cell surface triggers the classical pyroptosis pathway, leading to the release of HMGB1, IL‐1β, and IL‐18. Noncanonical pathway: chemotherapy and CAR‐T treatment induce cell death mediated by noncanonical focal prolapse pathway. Granzyme‐mediated pathway: TNF‐α/TRADD pathway induced focal prolapse and its mechanism.

The nonclassical pathway of pyroptosis is mediated primarily by caspase‐4, 5, and 11. Activation of these caspases occurs upon stimulation of cells with bacterial LPS, whereby they bind directly to LPS and subsequently become activated.^[^ ^54^ ^]^ Upon activation, caspase‐4, 5, and 11 cleave GSDMD and remove the intramolecular inhibition of the GSDMD‐N structural domain.^[^ ^55^ ^]^ The liberated GSDMD‐N domain binds to cell membrane phospholipids, interacts with them, and causes pore formation, cell swelling, rupture of the membrane, and induction of pyroptosis.^[^ ^56^ ^]^ Additionally, the GSDMD‐N terminus activates the NLRP3 inflammasome, which in turn activates caspase‐1.^[^ ^56^ ^]^ Activation of caspase‐1 stimulates the maturation and secretion of IL‐18 and IL‐1β, thereby amplifying the inflammatory response.^[^ ^57^ ^]^ **Figure** **6** provides an overview of the molecular mechanisms involved in pyroptosis.

## 5 Ferroptosis in GI Cancers

### 5.1 Ferroptosis in the Proliferation, Invasion, and Metastasis of GI Cancers

Many ferroptosis‐associated molecules and signaling pathways can modulate the proliferation, invasion, and metastasis of GI cancers. There were several mechanistic studies exploring the role of ferroptosis in GI cancers (**Table** **1**). For example, erastin treatment of hepatocellular carcinoma (HCC) cells increased the expression of GABPB1‐AS1 and inhibited the expression of Peroxiredoxin 5, decreasing the antioxidant capacity of cells to enhance the malignant progression of HCC,^[^ ^58^ ^]^ while G6PD overexpression inhibited ferroptosis and promoted malignant progression in HCC cells and decreased ferroptosis in HCC cells by inhibiting Cytochrome P450 Oxidoreductase.^[^ ^59^, ^60^ ^]^ The YTHDF2/solute carrier family 7 member 11 (SLC7A11) regulatory axis could be a therapeutic target for HCC interventional embolization therapy to inhibit ferroptosis in HCC cells.^[^ ^61^ ^]^ Abnormal methylation of the promoter region of Protocadherin Beta 14 (PCDHB14) leads to its reduced expression in HCC cells,^[^ ^62^ ^]^ which can inhibit cell proliferation and induce ferroptosis in HCC cells, while rhamnolipids suppressed cell proliferation and aggression, and rhamnetin induced ferroptosis by inhibiting GPX4 expression.^[^ ^63^ ^]^ Moreover, HULC promoted HCC cell proliferation and metastasis, inhibited ferroptosis by binding to miR‐3200‐5b and targeting Activating transcription factor 4, and YAP‐ TEA Domain enhanced ferroptosis by targeting Arachidonate lipoxygenase 3, effectively increasing their vulnerability to ferritin‐mediated cell death.^[^ ^64^ ^]^

**Table 1:** Overview of targeted molecules and signaling pathways involved in the malignant progression of GI cancers associated with ferroptosis

Targets	Expression	Ferroptosis	Biological effects	Mechanism
GABPB1‐AS1	Up	Suppression	Inhibit antioxidant capacity	GABPB1‐AS1/GABPB1/PRDX5
CircIL4R	Up	Suppression	Promote malignant progression	CircIL4R/miR‐541‐3/GPX4
G6PD	Up	Suppression	Promote cell proliferation, migration, and invasion	G6PD/POR
METTL14	Up	Suppression	Promote malignant progress	HIF‐1α/METTL14/YTHDF2/SLC7A11
YAP	Up	Suppression	Promote malignant progress	YAP‐TEAD/ALOXE3
HULC	Up	Suppression	Promote cell proliferation and metastasis	HULC/miR‐3200‐5b/ATF4
PCDHB14	Down	Promotion	Block cell cycle and inhibit cell proliferation	PCDHB14/RNF182/SLC7A11
Rhamnosin	–	Promotion	Inhibit cell proliferation and invasion	Rhamnosin/GPX4
Gentian violet	–	Promotion	Inhibit cell proliferation, migration, and invasion, and induce cell apoptosis	Gentian violet/Hep27‐MDM2‐p53
Lnc‐HEPFAL	Down	Promotion	Inhibit malignant progression	Lnc‐HEPFAL/SLC7A11
CPLX2	Up	Suppression	Promote cell vitality, and inhibit and promote cell apoptosis	CPLX2/NRF2
OIT3	Down	Promotion	Inhibit cell proliferation, migration, and invasion	OIT3/ALOX15/CYP4F3
HDLBP	Up	Suppression	Promote malignant progress	HDLBP/lncFAL/FSP1
PNO1	Up	Suppression	Promote malignant progress	–
Anisomycin	–	Promotion	Inhibit the formation and migration of cell colonies and induce cell death	Anisamycin/p38MAPK/p‐H3S10
CircABCB10	Up	Suppression	Promote cell apoptosis	CircABCB10/miR‐326/CCL5
ACADSB	Down	Promotion	Inhibit cell migration, invasion, and proliferation	ACADSB/GSH reductase and GSH peroxidase 4
DCA	Down	Promotion	Promote malignant progress	–
SFRS9	Up	Suppression	Promote cell viability, cell cycle progression, and colony formation	SFRS9/GPX4
Propofol	–	Promotion	Inhibit cell proliferation and colony formation	Propofol/STAT3/GPX4
Genistein	–	Promotion	Promote cell apoptosis and oxidation, and significantly inhibit cell viability, invasion, and cloning	Genistein/Keap1/AIFM1
GCH1	–	Promotion	Induce apoptosis	GCH1/BH4
Pt3R5G	–	Promotion	Inhibit cell proliferation	Pt3R5G/SLC7A11
Lysionotin	–	Promotion	Inhibit cell proliferation, migration, and invasion	Lysionotin/Nrf2
DNAJB6	Up	Suppression	Promote malignant progress	DNAJB6a/GPX4/p‐AKT
5‐ALA	–	Promotion	Inhibit malignant progression	5‐ALA/GPX4/HMOX1
ARHGEF26‐AS1	Down	Promotion	Inhibit cell proliferation and migration	ARHGEF26‐AS1/miR‐372‐3p/ADAM23
BBOX1‐AS1	Up	Suppression	Promote cell proliferation, migration, and invasion, and inhibit cell apoptosis	BBOX1‐AS1/miR‐513a‐3p/SLC7A11
Allicin	–	Promotion	Inhibit cell proliferation	Allicin/AMPK/mTOR
perilipin2	Up	Suppression	Enhance cell proliferation and inhibit cell apoptosis	Ferroptosis pathway
ACPs	–	Promotion	Inhibit cell proliferation and migration	ACPs/GPX4/SLC7A11
SCD1	–	Suppression	Promote tumor growth and migration	Cancer stem cell cycle‐related protein
Levobupivacaine	Down	Promotion	Inhibit cell proliferation	Levobupivacaine/miR‐489‐3p/SLC7A11
CPEB1	Down	Promotion	Inhibit tumor growth	CPEB1/twist1/ATF4/CHAC1
Tanshinone IIA	–	Promotion	Inhibit tumor growth	Increase the level of lipid peroxide
Propofol	–	Promotion	Inhibit cell proliferation, migration, and invasion, and induction of cell apoptosis	Propofol/miR‐125b‐5p/STAT3
LTBP2	Up	Suppression	Promote cell proliferation	LTBP2/p62‐Keap1‐Nrf2
CST1	Up	Suppression	Promote cell migration and invasion, and increase tumor metastasis	CST1/GPX4/OTUB1
ATF2	Up	Suppression	Promote cell growth and metastasis	ATF2/HSPH1/SLC7A11

Treatment of HCC cells with gentian violet (GV) resulted in the inhibition of cell proliferation, migration, and invasion, and induced apoptosis, ferroptosis, and tumor growth inhibition. Ferrostatin‐1 and Z‐VAD‐KFM partially attenuated the GV‐induced growth inhibition, whereas GV regulated ferroptosis and apoptosis through the Hep27‐MDM2‐p53 signaling cascade.^[^ ^65^ ^]^ On the other hand, lncRNA‐HEPFAL expression was decreased in HCC tissues, and overexpression of lnc‐HEPFAL enhanced ferroptosis by increasing ROS and iron levels, improving ubiquitin‐mediated degradation of SLC7A11, thus increasing susceptibility to erastin‐induced ferroptosis.^[^ ^66^ ^]^ CPLX2 expression was increased in HCC and predicted poor prognosis in patients with HCC. Its inhibition decreased the viability of HCC cells and increased sorafenib‐induced cell death.^[^ ^67^ ^]^ cPLX2 stimulated nuclear factor E2‐related factor 2 (NRF2) expression and inhibited ferroptosis, thereby inhibiting HCC cell proliferation, migration, and invasion. Moreover, OIT3 overexpression inhibited the proliferation, migration, and invasion of HCC cells, while HDLBP could bind to lncFAL and stabilize its expression, reducing the vulnerability of cells to ferroptosis.^[^ ^68^ ^]^ Camptothecin treatment notably decreased HCC cell colony formation and migration, induced HCC cell death, and activated p38MAPK, thereby enhancing ferroptosis in HCC cells.^[^ ^69^ ^]^ Betula etnensis Raf. bark methanolic extract was shown to induce ferroptosis in CRC cells by increasing ROS levels, reducing RelA‐SpoT homologue levels, and increasing lipid peroxides and Heme Oxygenase‐1 expression. In Kirsten rat sarcoma viral oncogene homologue (KRAS)‐mutant CRC cells, combination treatment with β‐elemene and cetuximab promoted ferroptosis by alleviating the accumulation of reactive ROS, GSH depletion, and lipid peroxidation, upregulating HO‐1 and transferrin expression, and decreasing the expression of negative regulatory proteins related to ferroptosis. Furthermore, the combination of the two treatments inhibited cell migration, tumor growth, and lymph node metastasis.^[^ ^70^, ^71^ ^]^ The treatment of CRC cells with IMCA inhibited cell viability and tumor growth by inhibiting the expression of SLC7A11, decreasing the content of cysteine and GSH, leading to the accumulation of ROS and inducing ferroptosis. Additionally, IMCA regulated the activity of the AMPK/mTOR/p70S6k signaling pathway. It was found that circABCB10 expression was increased in rectal cancer cells and that it could inhibit ferroptosis and apoptosis. circABCB10 also regulated the miR‐326/C–C chemokine ligand 5 axis, which alleviated the malignant progression of rectal cancer.^[^ ^72^, ^73^ ^]^

Ferroptosis itself can also play an important role in regulating the progressions of different types of GI cancers. Many reported studies have convinced that the regulation of certain genes and proteins related to ferroptosis can inhibit or induce cell proliferation, migration, and invasion in GI cancers. For example, low expression of short/branched chain acyl‐CoA dehydrogenase (ACADSB) is predictive of poor patient prognosis in CRC, while overexpression of ACADSB inhibits the migration, invasion, and proliferation of CRC cells.^[^ ^74^ ^]^ Moreover, serine and arginine rich splicing factor 9 (SFRS9) markedly increases ferroptosis in CRC cells by inhibiting the expression of GPX4,^[^ ^75^ ^]^ which is necessary for the DCA‐mediated reduction of stemness and viability of CRC cells.^[^ ^76^ ^]^ In ESCC, ARHGEF26‐AS1 expression is reduced, and its binding to miR‐372‐3p enhanced the expression of anti‐leucine‐rich glioma‐inactivated 1 (LGI1) receptor A Disintegrin And Metalloprotease 23 (ADAM23), thereby promoting ferroptosis and inhibiting ESCC cell proliferation and migration. Finally, in GC cells, ATF2 inhibition reduced the growth and metastasis of cells and promoted sorafenib‐induced ferroptosis, while levobupivacaine induced ferroptosis and decreased the proliferation ability of GC cells by regulating the miR‐489‐3p/SLC7A11 axis.^[^ ^77^ ^]^ These studies suggested that increasing or decreasing ferroptosis activity could be a potential strategy for cancer treatment.

### 5.2 Ferroptosis in Tumor Microenvironment (TME) of GI Cancers

Several genes related to ferroptosis can modify the tumor microenvironment (TME) in GI cancers.^[^ ^93^, ^94^, ^95^, ^96^, ^97^ ^]^ miR‐1442‐3p expression was upregulated in hepatitis B virus (HBV)‐infected patients with hepatocellular carcinoma (HCC) and HBV‐infected M1 macrophages. Moreover, miR‐142‐3p downregulated SLC3A2, which promoted the proliferation, migration, and aggression of HCC cells and induced ferroptosis in HBV‐infected M1 macrophages.^[^ ^78^ ^]^ Glutamine synthase 2 (GLS2) played a significant role in promoting ferroptosis by increasing the conversion of glutamate to α‐ketoglutarate, consequently increasing lipid reactive oxygen species (ROS) production in HCC.^[^ ^79^ ^]^ Additionally, the expression of Apolipoprotein C1 (APOC1) was found to be substantially higher in tumor‐associated macrophages in HCC tissues than in normal tissues. Inhibition of APOC1 augmented the ferroptosis pathway, which facilitated the conversion of M2 to M1 macrophages, thereby remodeling the TME and improving the efficacy of anti‐PD1 immunotherapy in patients with HCC.^[^ ^80^ ^]^ Cetuximab treatment of KRAS‐mutant CRC cells intensified the cytotoxic effect of RSL3 on cells and enhanced RSL3‐induced ferroptosis by activating p38‐MAPK and suppressing the NRF2/HO‐1 axis.^[^ ^81^ ^]^ In GC, cancer‐associated fibroblasts secreted exosomal miR‐522, which targeted 12/15‐Lipoxygenase (ALOX15) and reduced lipid ROS accumulation by inhibiting ferritin formation in cancer cells. The USP7/HNRNP A1/exosomal‐miR‐522/ALOX15 signaling pathway provided a possible target for GC with acquired chemoresistance.^[^ ^82^ ^]^

### 5.3 Ferroptosis and Drug Resistance of GI Cancers

GI cancers’ drug resistance can be regulated by several ferroptosis‐related genes. Sorafenib treatment in HCC cells promotes ferroptosis, while NRF2 and S1R inhibit it by activating the transcription of quinone oxidoreductase‐1, heme oxygenase‐1, and ferritin weighty chain‐1. Ferroptosis can also be enhanced by inhibiting NRF2 and QSOX1 expression, promoting SPARC overexpression, and inhibiting GSTZ1 activity. The downregulation of GSTZ1 in sorafenib‐resistant HCC leads to a reduction in ferroptosis. In HCC, p62 inhibited the degradation of NRF2 and increased its nuclear accumulation by suppressing the activity of Kelch‐like ECH‐related protein 1. NRF2 interacted with MAF bZIP transcription factor G (MAFG) in the nucleus to activate the transcription of quinone oxidoreductase‐1, heme oxygenase‐1, and ferritin weighty chain‐1, thereby inhibiting erastin and sorafenib‐induced ferroptosis. In contrast, inhibition of NRF2 expression markedly enhanced the anticancer activity of erastin and sorafenib in HCC cells.^[^ ^83^ ^]^ In HCC cells, sorafenib treatment induced Sigma‐1 receptor (S1R) migration from the nucleus and upregulated the S1R protein expression; NRF2 inhibition notably increased the mRNA expression of S1R. S1R inhibition blocked the expression of GPX4, FTH1, and transferrin receptor protein 1, thereby inhibiting ferroptosis and promoting HCC malignant progression.^[^ ^84^ ^]^ Quiescin sulfhydryl oxidase 1 (QSOX1) acts as a cellular pro‐oxidant by inhibiting NRF2 activation, thereby enhancing the sensitivity of HCC cells to oxidative stress. Mechanistic studies have shown that QSOX1 decreased NRF2 activity and EGFR activation by promoting ubiquitination‐mediated EGFR degradation and accelerating its transport in intracellular endosomes. In addition, QSOX1 also enhanced sorafenib‐induced ferroptosis by inhibiting NRF2.^[^ ^85^ ^]^ Secreted protein acidic and rich in cysteine (SPARC) overexpression induced oxidative stress (release of lactate dehydrogenase (LDH) and ROS) leading to ferroptosis in HCC cells, which, in turn, enhanced the cytotoxic influence of sorafenib.^[^ ^86^ ^]^ Glutathione S‐transferase zeta 1(GSTZ1) enhanced sorafenib‐induced ferroptosis in HCC cells by inhibiting the NRF2/GPX4 axis; notably, its expression was downregulated in sorafenib‐resistant HCC cells.^[^ ^87^ ^]^

Disulfiram (DSF)/Cu selectively exerted potent cytotoxic effects and inhibited migration, aggression, and angiogenesis of HCC cells. DSF/Cu enhanced ferroptosis by impairing mitochondrial homeostasis, increasing free iron pools, and promoting lipid peroxidation. DSF/Cu activated the phosphorylation of p62 and increased its binding to Keap1, which prolonged the half‐life of NRF2. In addition, DSF/Cu enhanced the cytotoxicity of sorafenib and suppressed tumor growth by inhibiting both NRF2 and MAPK signaling pathways.^[^ ^88^ ^]^ CDGSH Iron Sulfur Domain 2 (CISD2) expression was increased in HCC cells and indicated poor patient prognosis. Knockdown of CISD2 decreased the viability of drug‐resistant HCC cells and increased the levels of ROS, MDA, and ferric ions. Moreover, CISD2 suppressed sorafenib‐induced ferroptosis in drug‐resistant HCC cells by inhibiting Beclin1‐related cellular autophagy.^[^ ^89^ ^]^ Lenvatinib has been approved for the treatment of advanced HCC; however, the underlying mechanism is not clear. Recently, Moffitt et al. reported that inhibition of NRF2 increased lenvatinib sensitivity and lipid ROS levels in HCC cells.^[^ ^90^ ^]^ Further, estrogen‐related receptor (ERR) expression was remarkably increased in HCC tissues, which was associated with increased cell proliferation. Treatment with ERRγ inverse agonist, DN200434, increased mitochondrial ROS production and decreased mTOR activity and GPX4 levels in HCC cells by decreasing GSH/GSH disulfide levels, which, in turn, increased ferroptosis and their sensitivity to sorafenib.^[^ ^91^ ^]^

Furthermore, Kinesin family member 20A (KIF20A) expression was increased in oxaliplatin‐resistant CRC cells, and its inhibition remarkably enhanced the sensitivity of the cells to oxaliplatin. KIF20A inhibited ferroptosis and induced oxaliplatin resistance in CRC cells by regulating the NUAK family kinase 1 (NUAK1)/PP1β/GPX4 signaling axis.^[^ ^92^ ^]^ Overexpression of FAM98A increased cell proliferation and decreased ferroptosis by activating the translation of SLC7A11 in stress granules. Moreover, metformin treatment reversed the FAM98A‐mediated resistance to 5‐FU in CRC cells.^[^ ^93^ ^]^ Oxaliplatin treatment reduced cell viability and induced ferroptosis and oxidative stress in CRC cells by inhibiting the NRF2 signaling pathway.^[^ ^94^ ^]^ Treatment of CRC cells with adipose‐derived exosomes reduced cellular sensitivity to ferritin and increased chemoresistance to oxaliplatin. Mechanistic tests showed that adipose‐derived exosomal microsomal triglyceride transfer protein (MTTP) downregulated ZEB1 expression by binding to PARP1 and upregulated GPX4 and xCT, thereby contributing to a decrease in n‐6/n‐3 polyunsaturated fatty acid ratio and lipid ROS levels.^[^ ^95^ ^]^ In GC, apatinib induced lipid peroxidation through Sterol regulatory element binding protein (SREBP)−1a‐mediated GPX4, thereby increasing ferroptosis in multidrug‐resistant GC cells.^[^ ^96^ ^]^ ATF3 expression was reduced in GC tissues and cells, and high ATF3 expression predicted a better prognosis for patients with GC. Mechanistic studies suggested that ATF3 induced ferroptosis by blocking NRF2/Keap1/xCT signaling, consequently increasing cisplatin sensitivity in GC cells.^[^ ^97^ ^]^ Inhibition of Sirtuin 6 (SIRT6) expression promoted sorafenib sensitivity in GC cells by increasing ferroptosis. SIRT6 inhibited the malignant progression of GC by activating the Keap1/NRF2 signaling pathway and upregulating the expression of GPX4.^[^ ^98^ ^]^ Further, GC cells enhanced their SCD1 expression by secreting exo‐lncFERO into GC stem cells (GCSCs), which inhibited the expression of ferritin by directly interacting with SCD1 mRNA or by recruiting hnRNPA1. Moreover, hnRNPA1 was also involved in the packaging of lncFERO into exosomes in GC cells; upregulation of hnRNPA1 expression in GCSCs induced GC cells to secrete lncFERO, which increased tumor stemness and chemoresistance.^[^ ^99^ ^]^

### 5.4 Nanomaterials Targeting Ferroptosis for GI Cancers Treatment

Multiple novel nanomaterials have demonstrated the ability to target and impede the progression of GI cancers through the modulation of ferroptosis. The one‐pot synthesized manganese‐doped HCC successfully heightened ferroptosis within tumor cells by degrading intracellular glutathione (GSH) levels, eventually leading to GSH depletion. Sorafenib loaded in manganese silica nanoparticles (MMSNs) (MMSNs@SO) was utilized in the treatment of HCC cells; the resultant nanoformulation induced ferroptosis by reducing GSH and suppressing intracellular GSH synthesis. As a consequence of this action, the glutathione peroxidase 4 (GPX4) was inactivated, causing an increase in intracellular lipid peroxidation.^[^ ^100^ ^]^ Specifically, poly‐pyridine (PPy) nanoparticles combined with magnetite (Fe3O4) demonstrated the capacity to diminish the growth, migration, and invasion of colorectal cancer (CRC) cells by promoting ferroptosis. Further analysis indicated that these nanoparticles subdued the malignancy of CRC by the regulation of the nuclear factor kappa light‐chain enhancer of activated B cells (NF‐κB) signaling pathway.^[^ ^101^ ^]^ Ophiopogon B was capable of provoking ferroptosis in GC cells by preventing the GPX4/xCT system.^[^ ^102^ ^]^ Self‐assembled nanoparticles accelerated the process of lipid peroxidation by increasing the depletion of GSH and stimulating SO2 production. Additionally, they hampered the malignant development of GC by suppressing GPX4 expression and allowing ferroptosis to occur.^[^ ^103^ ^]^

### 5.5 Associations of Ferroptosis and Radiotherapy in GI Cancers

Ferroptosis‐associated genes have the capacity to modulate radiosensitivity in GI cancer cells. Within HCC cell lines, copper is known to play a crucial role in promoting radiation resistance. At the molecular level, ionizing radiation (IR) has been identified as an inhibitor of copper metabolism MURR1 domain 10 (COMMD10) expression levels. This inhibition subsequently instigates an increase in intracellular copper, further inducing radiation resistance in HCC. Mechanistically, IR‐induced low expression of COMMD10 inhibited ubiquitin‐mediated degradation of HIF‐1α, resulting in increased nuclear translocation of HIF‐1α and transcription of copper cyanine and SLC7A11. Elevated levels of copper cyanine decrease Fe, ultimately forming a positive feedback loop that increases HIF‐1α expression. Ultimately, COMMD10 enhances ferroptosis and radiosensitivity by disrupting Cu/Fe homeostasis in HCC.^[^ ^104^ ^]^

Further research demonstrates that Suppressor of cytokine signaling 2 (SOCS2) has the potential to increase the radiosensitivity of HCC. Additionally, ferroptosis has also been recognized as increasing the sensitivity of HCC to radiotherapy. SOCS2 improves HCC radiotherapy efficacy by promoting the ubiquitin‐mediated degradation of SLC7A11 and increasing ferroptosis.^[^ ^105^ ^]^ A marked increase in stearoyl‐CoA desaturase 1 (SCD1) expression levels was detected in esophageal squamous cell carcinoma (ESCC), and this elevated expression was deemed a predictor of poor patient prognosis. Treatment with SCD1 decreased ESCC cell ferroptosis and conferred radiation resistance by promoting the biosynthesis of oleic and palmitoleic acid, whereas the use of MF‐438, an SCD1 inhibitor, enhanced ferroptosis and immunogenic cell death in tumor cells, increasing the efficacy of radiotherapy in ESCC.^[^ ^106^ ^]^ Of note, the expressions of SLC7A11 in ESCC tissues and NRF2 in the nucleus were directly related to the prognosis of patients with ESCC. Taken together, it becomes apparent that the NRF2/SLC7A11/ferroptosis axis could be a suitable therapeutic target for ESCC.^[^ ^107^ ^]^

### 5.6 Ferroptosis‐Related Genes in GI Cancers

We further try to introduce the ferroptosis‐related genes in GI cancers, and hope these findings could contribute to clinical treatment of GI cancers. Treatment of CRC cells with a ferritin inducer, RSL3, contributed to an increase in ROS levels and transferrin expression along with a decrease in GPX4 expression, which, in turn, induced ferroptosis in CRC cells.^[^ ^108^ ^]^ Overexpression of Serine/arginine‐rich splicing factor 9 (SRSF9) in CRC cells decreased erastin‐induced ferroptosis and increased lipid peroxidation damage. Mechanistically, SRSF9 inhibited erastin‐induced ferroptosis and thus drives tumorigenesis by enhancing GPX4 levels.^[^ ^109^ ^]^ Cytoglobin treatment of CRC cells increased ROS accumulation and disrupted mitochondrial function, thereby increasing ferroptosis in CRC cells. Cytoglobin enhanced ferroptosis by regulating the p53‐YAP1/ACSL4 axis, which, in turn, inhibited the malignant progression of CRC.^[^ ^110^ ^]^ Overexpression of lipocalin 2 in CRC cells induced 5‐FU resistance by inhibiting ferroptosis. Mechanistically, lipocalin 2 reduced intracellular iron levels and stimulated the expression of GPX4 and SLC7A11.^[^ ^111^ ^]^ Treatment of CRC cells with tagitinin C inhibited cell growth, induced oxidation of the cellular microenvironment, and enhanced ferroptosis. Elesclomol suppressed the malignant progression of CRC by increasing the degradation of copper‐transporting ATPase 1 (ATP7A) and SLC7A11.^[^ ^112^ ^]^ Moreover, elesclomol inhibited CRC progression by enhancing cellular oxidative stress‐induced ferroptosis.^[^ ^113^ ^]^ miR‐15a‐3p increased the ROS, intracellular Fe^2+^ levels, and MDA accumulation by targeting GPX4. In addition, miR‐15a‐3p increased the sensitivity of CRC cells to erastin and GPX4 and thus induced ferroptosis.^[^ ^114^ ^]^ Overexpression of miR‐545 in CRC cells inhibited the expression of MDA, ROS, and Fe^2+^ and reversed the erastin‐ and RSL3‐induced decrease in CRC cell viability. Mechanistic studies indicated that miR‐545 targets transferrin to induce ferroptosis in CRC cells.^[^ ^115^ ^]^

GCH1 inhibited the release of BH4 and assisted in erastin‐induced cell death; it also enhanced lipid peroxidation and ferrous iron accumulation. Cotreatment of CRC with GCH1 inhibitors and erastin inhibited tumor growth. Therefore, aiming for GCH1/BH4 metabolism could increase erastin‐induced ferroptosis and thus limit CRC progression by activating iron phagosomes.^[^ ^116^ ^]^ TP53‐induced glycolysis and apoptosis regulator (TIGAR) expression was remarkably increased in CRC tissues, and its inhibition downregulated the GSH/glutathione disulfide (GSSG) ratio, increased lipid peroxidation, enhanced MDA accumulation, and promoted erastin‐induced ferroptosis. Overall, TIGAR inhibited ferroptosis and accelerated the malignant progression of CRC by regulating the ROS/AMPK/SCD1 signaling pathway.^[^ ^117^ ^]^ Subject Mediates the resistance of CRC cells to erastin‐induced ferroptosis to enhance tumor progression.^[^ ^118^ ^]^ In CRC cells carrying oncogenically activated PIK3CA, aspirin increased RSL3‐induced ferroptosis by inhibiting the AKT/mTOR signaling pathway and REBP‐1 expression, and attenuating SCD1‐mediated adipogenesis of monounsaturated fatty acids.^[^ ^119^ ^]^ LINC00239 was highly expressed in CRC tissues, and its high expression predicted poor prognosis in patients with CRC. LINC00239 (nucleotides 1–315) interacted with the Keap 1 (Kelch structural domain; NRF2 binding site); this interaction inhibited NRF2 ubiquitination and increased its stability, which, in turn, inhibited ferroptosis.^[^ ^120^ ^]^ circPVT1 expression was markedly increased in 5‐FU‐resistant ESCC cells; its knockdown increased cytotoxicity by downregulating multidrug resistance‐associated proteins such as P‐gp and multidrug resistance‐associated protein 1 (MRP1). circPVT1 increased the expression of Frizzled3 (FZD3) through sponging miR‐30a‐5p. In addition, circPVT1 inhibited ferroptosis by upregulating the expression of p‐β‐catenin, GPX4, and SLC7A11.^[^ ^121^ ^]^ Activation of Hsp27 and upregulation of GPX4 inhibited ferroptosis and alleviated the damage caused by elevated lipid peroxidation in CSCs. Therefore, targeting Hsp27 and GPX4 may inhibit the activity of CSCs and further suppress the malignant progression of ESCC.^[^ ^122^ ^]^ In GC cells, cysteine dioxygenase‐1 inhibited erastin‐induced ferroptosis by promoting GSH release and inhibiting MDA production.

Tan IIA induced ferroptosis by increasing lipid peroxidation and upregulating Ptgs2 and Chac1 expression. c‐Myb also promotes p53 expression and ROS release, and inhibited xCT, GSH, and cysteine expression.^[^ ^123^ ^]^ Tan IIA inhibited the malignant progression of GC by upregulating p53 expression, which mediates ferroptosis.^[^ ^124^ ^]^ miR‐375 expression was reduced in GC, which diminished GC cell stemness by targeting SLC7A11 to enhance ferroptosis.^[^ ^125^ ^]^ BDNF‐AS was highly expressed in GC, and its high expression was associated with poor prognosis in patients with GC. Overexpression of BDNF‐AS inhibited ferroptosis in GC cells and promoted malignant tumor progression. BDNF‐AS inhibited ferroptosis by regulating the WD40 repeat domain protein 5 (WDR5)/F‐box and WD repeat domain containing 7 (FBXW7) axis and increasing the ubiquitination of Voltage Dependent Anion Channel 3 (VDAC3).^[^ ^126^ ^]^ LncRNA‐PMAN was highly expressed in GC with peritoneal metastases, and its high expression predicted poor prognosis in patients with GC. Mechanistic findings suggested that HIF‐1α increased PMAN expression, which further enhanced the stability of SLC7A11 mRNA by enhancing ELAV like RNA binding protein 1 (ELAVL1) entry into the cytoplasm, increasing GSH levels, and inhibiting ROS and iron accumulation in cells. Overall, the HIF‐1α/PMAN/ELAVL1 regulatory axis promoted GC development and metastasis by inhibiting ferroptosis.^[^ ^127^ ^]^ STAT3 has been shown to bind to and regulate the expression of consensus DNA response elements in the promoters of FNR‐related genes (GPX4, SLC7A11, and FTH1) to inhibit ferroptosis and induce 5‐FU resistance.^[^ ^128^ ^]^ Hypoxic conditions in GC promoted the expression of lnc‐CBSLR, which interacted with YTHDF2 and reduced the stability of CBS mRNA by enhancing the binding of YTHDF2 to the m6A site of CBS. In addition, inhibition of CBS expression in GC cells decreased the methylation of ACSL4 protein and induced its polyubiquitination and degradation, thereby inducing resistance to ferroptosis.^[^ ^129^ ^]^ Activation of the Wnt/beta‐catenin signaling pathway in GC cells decreased the production of lipid ROS and inhibited ferroptosis. Mechanistic studies indicated that the beta‐catenin/TCF4 transcriptional complex bound directly to the promoter region of GPX4 and increased its transcription.^[^ ^130^ ^]^ XN4 promoted ferroptosis in GC and inhibited the malignant progression of GC by decreasing GPX4 and increasing NADPH oxidase 4 (NOX4) and ferritin PTGS2 expressions.^[^ ^131^ ^]^ Inhibition of transient receptor potential melastatin 2 (TRPM2) expression in GC cells strengthened erastin‐ and RSL3‐induced ferroptosis by reducing the stability of HIF‐1α and NRF2 proteins.^[^ ^132^ ^]^ All these above findings have convinced that there must be a regulation network of ferroptosis in GI cancers, and further to identify the key regulators of ferroptosis network in GI cancers may be the vital issue.

## 6 Role of Necroptosis in GI Cancers

### 6.1 Necroptosis and Proliferation, Invasion, and Metastasis of GI Cancers

Several signaling pathways and necroptosis‐mediated molecules play a critical role in the progression, invasion, and metastasis of GI cancers (**Table** **2**). Induction of apoptosis and necroptosis through modulation of Bcl‐2/caspase family members and RIP1/RIP3 protein expression led to a marked reduction in the growth and viability of Hep3B cells by Fenofibrate.^[^ ^133^ ^]^ A study revealed that miR‐675 supplemented by the first exon of H19 mRNA, aimed at FADD to enhance cell necroptosis, leading to cell death, thereby obstructing HCC progression.^[^ ^134^ ^]^ Overexpression of Heparinase (HPSE) in HCC tissues indicated poor prognosis of HCC patients. Further, in vitro coculture assays demonstrated that HPSE promoted transendothelial migration of HCC cells and induced necroptosis. In vivo tests showed striking results, indicating that HPSE increased the percentage of necroptosis in the HUVEC cell line and facilitated intrahepatic metastasis of tumors. Apigetrin, on the other hand, induced apoptosis and necroptosis in HCC cells, leading to G2/M phase cell cycle arrest and inhibition of growth and proliferation in Hep3B cells. The compound upregulated TNF expression and downregulated p‐p65 and IκB phosphorylation and Bcl‐xl expression, whereas Bax expression was elevated to induce necrosis markers RIP3, p‐RIP3, and p‐MLKL expression. Moreover, the upregulation of cleaved PARP and Caspase‐3 further confirmed apoptosis induction in apigetrin‐treated Hep3B cells. The findings of these studies suggested a promising therapeutic application of these molecules in the treatment of GI cancers.^[^ ^135^ ^]^

**Table 2:** Overview of targeted molecules and signaling pathways involved in the malignant progression of GI cancers associated with necroptosis

Targets	Expression	Necroptosis	Biological effects	Mechanism
Fenofibrate	–	Promotion	Inhibit cell growth and vitality	Fenofibrate/Bcl‐2/caspase/RIP1/RIP3
miR‐675	–	Promotion	Induce cell death	miR‐675/FADD
HPSE	Up	Suppression	Promote malignant progress	HPSE/SDC‐1/TNF‐α
Apigetrin	Up	Suppression	Promote cell apoptosis	Apigetrin/TNF/p‐p65/p‐IκB
HNRNP A1	Up	Suppression	Promote proliferation, migration, and invasion, and promote tumor growth	HNRNP A1/P16INK4
SORD	Down	Promotion	Inhibit tumor growth and dryness	SORD/LDHA

The overexpression of heterogeneous nuclear ribonucleoprotein A1 (HNRNP A1) has been observed to enhance the proliferation, migration, and invasion of HCC cells, promoting tumor growth, and delaying senescence. As evaluated through mechanistic tests, silence of HNRNP A1 has been found to promote the expression of P16INK4, causing cell cycle arrest. Additionally, regulation of necroptosis and mitochondrial dynamics by HNRNP A1 has been established.^[^ ^136^ ^]^ In HCC tissues, a reduction in sorbitol dehydrogenase (SORD) expression has been noted, which has been linked to poor overall survival in patients with HCC. SORD has been observed to suppress tumor growth and stemness via regulation of lactate dehydrogenase A (LDHA) expression and mitochondrial dynamics, which in turn enhances necroptosis. Treatment with human recombinant SORD has demonstrated inhibition of HCC cell growth as well as macrophage polarization.^[^ ^137^ ^]^ In colorectal cancer (CRC) cell lines, truncation of DATE has been shown to activate hepatocyte growth factor (HGF) expression, thereby reducing receptor‐interacting protein kinase 1 (RIPK1) expression through mesenchymal epithelial transition (MET) signaling. This subsequently promotes cell proliferation and inhibits necroptosis.^[^ ^138^ ^]^ By inducing necroptosis in vivo, bufadienolide has been observed to inhibit the growth and metastasis of CRC. Resibufogenin has been found to inhibit the growth and metastasis of CRC by upregulating RIPK3 expression and enhancing the phosphorylation of serine residue 358 of mixed lineage kinase domain‐like protein (MLKL), thereby activating O‐GlcNAcylation of liver glycogen phosphorylase (PYGL), glutamate delta receptors 1 (GLUD1), and glutamate‐ammonia ligase (GLUL) expression in a RIPK3‐dependent manner. Each of these cellular events enhances necroptosis in CRC cells. Additionally, resibufogenin displays cytotoxic effects by inducing the accumulation of ROS.^[^ ^139^ ^]^

In CRC, upregulation of glycolipid transfer protein (GLTP) expression has been shown to restrain cell growth by regulating key cell cycle regulatory genes. Notably, GLTP overexpression has been reported to activate Kip1/p27 and Cip1/p21 leading to the downregulation of cyclin‐dependent kinase 2 (CDK2), CDK4, cyclin E, and cyclin D1. GLTP overexpression has also been shown to induce necroptosis in CRC cells. This was indicated by the RIPK3‐mediated phosphorylation of pMLKL, elevated cytosolic calcium, and enhanced plasma membrane permeabilization due to pMLKL oligomerization.^[^ ^140^ ^]^ Radiotherapy has been found to promote necroptosis in CRC cells through the activation of the RIP1/RIP3/MLKL/JNK/IL‐8 pathway, which effectively blocks tumor repopulation. Furthermore, inhibition of SET and MYND domain‐containing protein 2 (SMYD2) expression has been shown to inhibit CRC tumor growth and promote TNF‐induced apoptosis and necroptosis. SMYD2 targets RIPK1 and inhibits its phosphorylation.^[^ ^141^ ^]^ Additional investigations into the use of nanotropic analogs have indicated promising outcomes. The HUHS1015 analog was found to reduce the viability of MKN28 and MKN45 cells (GC cell lines). HUHS1015 inhibited the cell cycle and enhanced necroptosis, leading to nuclear localization of AMID and induced nuclear division and agglutination. Treatment with Nec‐1 attenuated these biological effects.^[^ ^142^ ^]^

### 6.2 Necroptosis and Drug Resistance in GI Cancers

Several studies suggest that necroptosis‐related genes may play a crucial role in determining drug resistance of GI cancers. For instance, in HepG2/DDP cells, ectopic expression of RIP3 appeared to induce cellular necroptosis in response to cisplatin‐induced stress by modulating the RIP1‐RIP3‐MLKL signaling pathway. This effect was accompanied by the release of high mobility group box‐1 protein (HMGB1) and lactate dehydrogenase (LDH).^[^ ^143^ ^]^ In hypoxic conditions, heat shock protein 90 (HSP90) was found to promote sorafenib resistance by facilitating chaperone‐mediated autophagic degradation of MLKL, leading to a reduction in necroptosis. This phenomenon could be reversed with the use of demethoxygeldanamycin, a HSP90 inhibitor.^[^ ^144^ ^]^ In CRC cells, pan‐caspase inhibitors were found to promote 5‐fluorouracil (5‐FU)‐induced necroptosis by increasing the autocrine secretion of TNF‐α. The combination of a novel pan‐caspase inhibitor, IDN‐7314, with 5‐FU, showed promising results in inhibiting CRC cell growth.^[^ ^145^ ^]^ In CRC, the overexpression of miR‐29b‐3p was found to be involved in resistance to 5‐FU‐induced necroptosis by targeting TRAF5.^[^ ^146^ ^]^ In ESCC, high expression of STAT3 was found to correlate with better survival and enhanced sensitivity to concurrent chemoradiotherapy. Mechanistic tests found an upregulation of the TNF signaling pathway and necrotic cell death pathway‐related protein expression in CCRT‐treated high‐STAT3‐expressing cells, suggesting a direct link between STAT3 and necroptosis.^[^ ^147^ ^]^ Finally, in GC cells, the combination of the antidepressant fluoxetine with paclitaxel was found to induce apoptosis and necroptosis and block cell cycle progression in the G2/M phase, leading to enhanced antiproliferative effects.^[^ ^148^ ^]^

### 6.3 Nanomaterials Targeting Necroptosis to Treat GI Cancers

There is a discrepancy in the literature with regards to the impact of nanomaterials on cell necrosis. While some studies have focused on the reduction of cellular viability without considering the specific type of cell death, others have identified instances of secondary necrosis following apoptosis, leading to potentially inaccurate conclusions.^[^ ^149^ ^]^ Prior research has exhibited the ability of nanomaterials to regulate necroptosis as a means of modulating the progression of gastrointestinal cancers. For example, VV‐SMAC is a Vaccinia virus that targets tumors and contains the SMAC/DIABLO gene, which has been knocked out in the thymidine kinase gene region. VV‐SMAC destroys HCC cells through caspase‐dependent apoptosis and necrosis, and by consuming apoptotic protein inhibitors. Furthermore, amalgamating VV‐SMAC with vinblastine leads to lysosome formation and HCC cell death.^[^ ^150^ ^]^ In another study, the treatment of HCC cells with silicon dioxide nanoparticles (nano‐SiO_2_) led to an increase in cell cycle arrest, apoptosis, and necroptosis. Nano‐SiO_2_ promoted the expression of the Z‐DNA binding protein‐1 (ZBP‐1), thereby inducing necroptosis.^[^ ^151^ ^]^ Additionally, an alkyl gold(I) complex [Au(C^C‐2‐NC5H4)(PTA)] was developed as a strategy to inhibit colorectal cancer progression in vitro. The complex induced necroptosis by enhancing the release of ROS.^[^ ^152^ ^]^ Finally, nanoparticles were found to be effective in inhibiting the proliferation of HepG2 cells, and this phenomenon was attributed to the induction of apoptosis and necrosis through the endoplasmic reticulum stress.^[^ ^153^ ^]^ This evidence highlights the potential future clinical application of nanomaterials which can target necroptosis.

### 6.4 Necroptosis‐Related Genes in GI Cancers

In HCC cells, Tan IIA downregulated the expression of the short form of FLICE inhibitory protein (FLIP) and led to the formation of homodimers of cleaved caspase‐8, which induced apoptosis by cleaving RIP1, RIP3, and MLKL. In contrast, Nec‐1 restored FLIP expression (which was downregulated by Tan IIA) and promoted the formation of heterodimers between FLIP and caspase‐8, thereby preventing apoptosis. Overall, Tan IIA induced both Nec‐1 inhibition and FLIP regulation‐mediated apoptosis and necroptosis.^[^ ^154^ ^]^ Inhibition of p66shc expression or promotion of SerpinB3 expression enhanced necroptosis and thus suppressed the malignant progression of HCC.^[^ ^155^ ^]^ Connexin32 (Cx32) expression was increased in HCC tissues and cell lines, and positively correlated with the expression of necrosis protein biomarkers in xenograft models. Cx32 interacted with Src and enhanced Src‐mediated caspase‐8 phosphorylation and inactivation, thereby inhibiting RIP1‐RIP3 proteolysis.^[^ ^156^ ^]^ Dimethyl fumarate induced necroptosis in colon cancer cells by depleting GSH, increasing ROS levels, and activating MAPKs.^[^ ^157^ ^]^ Colon cancer cells treated with 2‐methoxy‐6‐acetyl‐7‐methyljusqualone (MAM) demonstrated the formation of a RIP1/RIP3 complex to promote necroptosis. This complex triggered necroptosis through cytosolic Ca^2+^ accumulation and sustained JNK activation.^[^ ^158^ ^]^

Some investigators further purified a cationic peroxidase from raw millet seeds and evaluated its effect on CRC cells. The peroxidase inhibited malignant tumor progression by inducing necroptosis through the autocrine production of TNF‐α and restoration of RIPK3 expression.^[^ ^159^ ^]^ Fragile X mental retardation protein (FMRP) expression was notably increased in CRC tissues, and its inhibition induced necroptosis, thereby limiting carcinogenesis and progression. FMRP interacted with RIPK1 and increased its expression.^[^ ^160^ ^]^ In CRC, Phosphofructokinase‐15 (PFK‐15) induced necroptosis by increasing phosphorylation of RIP1, RIP3, and MLKL and further enhancing RIP3 and RIP1/MLKL interactions.^[^ ^161^ ^]^ Ergothioneine treatment of CRC cells induced the accumulation of ROS, loss of mitochondrial membrane potential, and increased SIRT3 expression. Ergothioneine promoted the interaction between MLKL and SIRT3, and thus induced necroptosis in CRC cells.^[^ ^162^ ^]^ Besides, treatment of CRC cells with demulsified whey downregulated the protein expression levels of c‐myc, phospho‐histone H3 (ser 10), and p‐ERK and induced necroptosis by activating the RIPK1, RIPK3, and MLKL signaling axis.^[^ ^163^ ^]^ The RIP3/MLKL signaling pathway and downregulation of Biglycan (BGN) inhibited the levels of proinflammatory cytokines in HGC‐27 and AGS cells and induced necroptosis.^[^ ^164^ ^]^ Astaxanthin notably promoted necroptosis in GC cells by activating nicotinamide adenine dinucleotide phosphate (NADPH) oxidase and RIP1/RIP3/MLKL signaling.^[^ ^165^ ^]^ These findings also demonstrated the regulatory network of necroptosis in GI cancers. Furthermore, the above‐mentioned molecules and signaling pathways can be considered as potentially effective targets for therapeutic strategy, given the critical role of necroptosis in suppressing the progression of GI cancers.

## 7 Pyroptosis in GI Cancers

### 7.1 Pyroptosis and Proliferation, Invasion, and Metastasis of GI Cancers

Various molecules and signaling pathways associated with pyroptosis have been implicated in modulating the proliferation, invasion, and metastasis of GI cancers (**Table** **3**). Notably, treatment of HCC cells with alpinumisoflavone inhibited cell proliferation, migration, and invasion by enhancing pyroptosis through its induction of NLRP3 inflammasome‐mediated autophagy.^[^ ^166^ ^]^ Miltirone also decreased the cell viability of HCC cells via proteolytic cleavage of gasdermin E (GSDME) and caspase‐3. Knockdown of GSDME switched miltirone‐induced cell death from pyroptosis to apoptosis, while caspase‐3 silencing attenuated GSDME‐dependent pyroptosis by miltirone. The induction of pyroptosis by miltirone is attributed to the increased accumulation of ROS and the inhibition of MEK and ERK1/2 phosphorylation.^[^ ^167^ ^]^ Furthermore, cannabidiol (CBD) demonstrated a caspase‐3/GSDME‐dependent inhibitory effect on HCC cell growth, inducing pyroptosis by enhancing integrated and mitochondrial stress responses. The Akt regulatory axis modulated the suppressive impact of CBD‐induced aerobic glycolysis on HCC cells.^[^ ^168^ ^]^ Metformin also induced pyroptosis in HCC cells by upregulating forkhead box O3 (FOXO3) expression, which activated NLRP3 transcription to inhibit cell proliferation and promote apoptosis.^[^ ^169^ ^]^ Inhibition of NIMA (never in mitosis gene a)‐related kinase 7 (NEK7) inhibited hepatic stellate cell activation in HCC by suppressing NLRP3, caspase‐1, and GSDMD expression, leading to reduced cell viability, migration, invasion, and tumor growth.^[^ ^170^ ^]^

**Table 3:** Overview of targeted molecules and signaling pathways involved in the malignant progression of GI cancers associated with pyroptosis

Targets	Expression	Pyroptosis	Biological effects	Mechanism
AIF	–	Promotion	Inhibition of cell proliferation, migration, and invasion	AIF/NLRP3
Miltirone	–	Promotion	Inhibit cell viability	Promote the accumulation of ROS and inhibit the phosphorylation of MEK and ERK1/2
CBD	–	Promotion	Inhibit cell growth	ATF4‐IGFBP1‐Akt
Met	–	Promotion	Suppress cell proliferation, promote cell apoptosis, and inhibit tumor growth	Met/FOXO3/NLRP3
NEK7	Up	Suppression	Promote cell viability, migration, invasion, and tumor growth	NEK7/NLRP3/Caspase‐1/GSDMD
MLD	–	Promotion	Inhibit cell proliferation, DNA synthesis, and colony formation	MLD/TOM20
Lobaplatin	–	Promotion	Suppress malignant process	Lobaplatin/caspase‐3/GSDME
FL118	–	Promotion	Inhibit cell proliferation, migration, and invasion	FL118/NLRP3‐ASC‐Caspase‐1
SDG	–	Promotion	Inhibit cell growth	ROS/P13K/AKT/BAK‐mitochondrial apoptosis

Meanwhile, the activation of pyroptosis and autophagy pathways induced by mallotucin D, which caused mitochondrial damage and ROS release, was inhibited by using N‐acetylcysteine (NAC), an ROS scavenger. This pathway was promoted by the migration of cytochrome c from mitochondria to the cytoplasm, which caused caspase‐9 and caspase‐3 to cleave GSDMD and induce pyroptosis. Mallotucin D activation of mitophagy was also inhibited by NAC through PI3K/AKT/mTOR pathway suppression.^[^ ^170^ ^]^ Knockdown of GSDME stimulated a shift from pyroptosis to apoptosis, whereas lobaplatin treatment elevated ROS levels and phosphorylation of c‐Jun N‐terminal kinase (JNK), activating Bax recruitment to the mitochondria and stimulating the cleavage of cytochrome c into the cytoplasm for the induction of pyroptosis.^[^ ^171^ ^]^ In CRC cells, FL118 induced pyroptosis through the NLRP3‐ASC‐caspase‐1 pathway, inhibiting cell proliferation, migration, and invasion, while SDG‐induced pyroptosis was dependent on ROS/PI3K/AKT/BAK‐mitochondrial apoptosis pathway, causing GSDMD cleavage and decreased malignant progression of CRC.^[^ ^172^, ^173^ ^]^ These findings underscore the crucial role of pyroptosis as a mechanism in the anticancer activity of various therapeutic agents.

### 7.2 Pyroptosis and TME Modulation in GI Cancers

Pyroptosis, a highly inflammatory PCD, offers a distinct advantage over cell apoptosis, which is commonly regarded as an immune‐tolerant process, in promoting the systemic immune response to solid tumors and alleviating immune suppression.^[^ ^174^ ^]^ The activation of inflammatory caspase, which cleaves gasdermin D (GSDMD), represents a key event during pyroptosis and culminates in the release of gasdermin D N‐terminal domain (N‐GSDMD). Pyroptosis leads to the conversion of immunosuppressive “cold” TME into immunogenic “hot” TME and induces immunogenic cell death (ICD), resulting in an influx of tumor‐infiltrating lymphocytes.^[^ ^175^ ^]^ In addition, pyrolytic cells not only function as tumor‐associated antigens by releasing cell contents, consisting of cytokines and proinflammatory factors, but also serve as danger signals, releasing danger‐associated molecular patterns (DAMPs) that act as immune adjuvants for the recruitment and maturation of antigen‐presenting cells (APCs). This, in turn, leads to stronger immune activation and regulatory effects on TME.^[^ ^176^ ^]^ The modulation of TME by pyroptosis‐related genes may contribute to the progression of GI cancers. Sorafenib, used as a treatment for HCC, increases pyroptosis in macrophages, activating cytotoxic NK cells and ultimately leading to tumor cell death. Sorafenib also inhibits the expression of major histocompatibility complex class I in HCC cells. However, the inhibition of IL‐1β and IL‐18 reduced the efficacy of sorafenib, thereby increasing pyroptosis.^[^ ^177^ ^]^ The treatment of CRC with gambogic acid (GA) increases the proportion of CD3^+^ T cells, cytotoxic T lymphocytes, dendritic cells, and effector memory T cells (CD8^+^, CD44^+^, and CD62L). GA induces caspase‐3/GSDME‐dependent pyroptosis and inhibits CRC progression.^[^ ^178^ ^]^ Thus, by triggering pyroptosis, chemotherapeutic agents can modulate TME, thereby enhancing the antitumor ability.

### 7.3 Pyroptosis and Drug Resistance in GI cancers

Numerous pyroptosis‐related genes can modulate drug resistance in GI cancers. GW4064 acted synergistically with oxaliplatin to inhibit CRC cell growth and colony formation by inducing apoptosis and pyroptosis. Mechanistic studies showed that GW4064 enhanced the sensitivity of CRC cells to oxaliplatin by inducing BAX/caspase‐3/GSDME‐mediated pyroptosis. In addition, the combination of oxaliplatin and GW4064 promoted SHP expression and inhibited STAT3 signaling.^[^ ^179^ ^]^ The expression of the deubiquitinating enzyme USP47 was reduced in CRC, and the knockdown of USP47 in CRC cells increased doxorubicin‐induced pyroptosis and apoptosis. USP47 binds to TCEA3 and regulates its deubiquitination and intracellular levels, thereby enhancing doxorubicin resistance in CRC cells.^[^ ^180^ ^]^ STAT3β treatment enhanced cisplatin sensitivity and GSDME‐dependent pyroptosis in ESCC cells; mechanistic tests showed that STAT3β treatment increased ROS levels in cisplatin‐treated cells, thereby activating caspase‐3 and GSDME and thus inducing pyroptosis. STAT3β interacted with ERK1/2 and blocked the phosphorylation of STAT3α S727 leading to GSDME activation.^[^ ^181^ ^]^ Treatment of GC cells with 5‐FU reduced cell viability and enhanced LDH release. In addition, 5‐FU treatment induced GSDME cleavage in GC cells, while knockdown of GSDME shifted 5‐FU‐induced pyroptosis to apoptosis in GC cells.^[^ ^182^ ^]^ Low concentrations of BIX‐01294 increased the sensitivity of GC cells to chemotherapy and markedly reduced cell viability. BIX enhanced the effect of chemotherapy by activating autophagic flux and inducing GSDME‐mediated pyroptosis in GC cells.^[^ ^183^ ^]^ These results indicated that pyroptosis greatly affect the therapeutic effect of various antitumor agents in GI cancers.

### 7.4 Nanomaterials Targeting Pyroptosis for GI Cancers Treatment

As a promising antitumor modality, regulating cell apoptosis and death pathways can effectively inhibit the proliferation and migration of cancer cells. Generally speaking, pyroptosis can be triggered by ROS induced by chemical drugs, but drug resistance and side effects severely limit its applications.^[^ ^184^ ^]^ Many researchers focused on more effective and noninvasive methods based on advanced nanotechnology, such as photodynamic therapy (PDT),^[^ ^185^ ^]^ nanocatalytic therapy,^[^ ^186^ ^]^ and photothermal therapy (PTT) to activate pyroptosis.^[^ ^187^ ^]^ Several new nanomaterials that target pyroptosis can inhibit the progression of GI cancers. A researcher synthesized the zeolite imidazole framework‐8 and encapsulated hydrophobic chlorin e6 and hydrophilic tirapazamine in it for synergistic acoustic chemotherapy and pyroptosis. Chlorin e6‐mediated sonodynamic therapy combined with ultrasound irradiation may aggravate hypoxia and thus activate tirapazamine, which may further exert antitumor effects by inducing pyroptosis in GC cells.^[^ ^188^ ^]^ Generally, these reported findings predicted that nanomaterial‐based drugs can be promising therapeutic agents in the treatment of GI cancers by targeting pyroptosis.

### 7.5 Pyroptosis‐Related Genes in GI Cancers

17β‐estradiol (E2) treatment promoted apoptosis and pyroptosis in HCC cells, which were reversed with caspase‐1 antagonist YVAD‐cmk. Moreover, E2 inhibited autophagy by activating the NLRP3 inflammasome through the ERβ/AMPK/mTOR pathway. Further, treatment with 3‐methyladenine enhanced E2‐induced cellular scorching. Overall, E2 induced activation of the NLRP3 inflammasome, which triggered pyroptosis and inhibited autophagy in HCC cells.^[^ ^189^ ^]^ Treatment of HCC cells with a branched‐chain polyunsaturated fatty acid, geranylgeranoic acid (GGA; C20:4), promoted Toll‐like receptor 4 (TLR4)‐mediated cellular autophagy, whereas treatment with VIPER, a specific inhibitor of TLR4 or knockdown of TLR4 reduced GGA‐induced cell death and mitochondrial unfolded protein response. GGA treatment enhanced NLRP3 and IL‐1β expression and NF‐κB entry into the nucleus. Therefore, GGA promoted pyroptosis in HCC cells by regulating TLR4.^[^ ^190^ ^]^ Small nucleolar RNA host gene 7 (SNHG7) expression was remarkably increased in HCC cells and tissues, which inhibited NLRP3‐dependent pyroptosis. Mechanistic studies showed that SNHG7 regulated SIRT1 through miR‐34a, which directly targets SIRT1. SNHG7 inhibition decreased SIRT1 expression but increased NLRP3, caspase‐1, and IL‐1β expression levels, thereby enhancing pyroptosis.^[^ ^191^ ^]^ Treatment of HCC cells with curcumin increased the expression of GSDME N‐terminus as well as other proteins involved in pyroptosis, and enhanced intracellular ROS levels, thereby increasing pyroptosis. N‐acetylcysteine treatment enhanced curcumin‐induced apoptosis and pyroptosis by inhibiting intracellular ROS production in HCC cells.^[^ ^192^ ^]^ Arsenic trioxide (ATO) and ascorbic acid (AA) inhibited the growth of CRC cells in a synergistic manner. AA and ATO coactivated caspase‐3 to trigger apoptosis and upregulated caspase‐1 expression to enhance inflammasome formation to induce pyroptosis, and ATO/AA can also induce the excessive release of ROS. Overall, the ATO/AA combination may be an effective treatment for CRC.^[^ ^193^ ^]^

The expression of GSDMD was remarkably reduced in CRC tissues, which indicated a poor prognosis for patients with CRC. LPS treatment enhanced CRC cell chemosensitivity to L‐OHP by improving GSDMD expression and GSDMD‐N membrane translocation. Therefore, LPS induced pyroptosis through the activation of GSDMD and enhanced the antitumor effect of L‐OHP.^[^ ^194^ ^]^ Treatment of CRC cells with miR‐21‐3p or miR‐21‐5p inhibitors as well as miR‐21‐5p mimics induced cell death. miR‐21‐5p enhanced pyroptosis by targeting TGFB1 and increasing the release of various inflammatory factors such as IL‐1β and IL‐18 and pyroptosis‐related mRNAs and proteins.^[^ ^195^ ^]^ IRs increased NEAT1 expression, which, in turn, increased GSDME expression by decreasing miR448 levels in CRC cells. NEAT1 inhibition decreased radiation‐induced pyroptosis as well as full‐length GSDME expression and GSDME cleavage to enhance CRC cell viability.^[^ ^196^ ^]^ Apoptin induced pyroptosis and GSDME cleavage by promoting caspase‐3 cleavage. When GSDME was reduced, apoptin induced pyroptosis. The GSDME‐dependent pyroptosis induced by apoptin was inhibited by using caspase‐3 and caspase‐9 inhibitors, and apoptin caused aggregation of the mitochondrial membrane protein Tom20 by increasing ROS levels.^[^ ^197^ ^]^ Besides, Patients with CRC having low FOXP2 expression had lower survival rates. In vitro cellular assays have shown that inhibition of FOXP2 upregulated the expression of proliferating cell nuclear antigen (PCNA) and cyclin D1, and downregulated the expression of caspase family proteins and GSDMD, thereby enhancing cell growth and inhibiting pyroptosis. Mechanistic studies have shown that FOXP2 interacts with caspase‐1 and promotes its expression.^[^ ^198^ ^]^ Metformin can inhibit tumor progression by inducing pyroptosis in ESCC by targeting the miR‐497/Proline‐, glutamic acid‐ and leucine‐rich protein 1 (PELP1) axis.^[^ ^199^ ^]^ Photodynamic therapy can treat ESCC by inducing GSDME‐mediated pyroptosis, and PDT can activate caspase‐8 and caspase‐3 via Pyruvate kinase Pyruvate kinase (PKM2) to ultimately release GSDME‐N and trigger pyroptosis in ESCC cells.^[^ ^200^ ^]^ Dihydroartemisinin (DHA) treatment reduced the expression of PKM2 and enhanced the production of caspase‐8/3 and GSDME‐NT as well as the release of LDH, IL‐18, and IL‐1β in ESCC cells. Therefore, DHA induced pyroptosis in ESCC cells through the PKM2/caspase‐8/3/GSDME pathway and inhibited the malignant progression of ESCC.^[^ ^201^ ^]^ We can imply from these reported results that targeted the key regulators of pyroptosis‐related regulatory network greatly affected the therapeutic effect of various antitumor agents in GI cancers and provided the potential targeted drug research and development directions.

## 8 Crosstalk between Ferroptosis, Necroptosis, and Pyroptosis in GI Cancers

Growing body of evidence indicated significant interplay among different forms of PCD including necrosis, pyroptosis, and ferroptosis. Per recent published studies, the initiation of necrosis was principally due to the involvement of macrophages. The opening of MLKL pore contributed to potassium efflux, activating NLRP3 inflammasomes.^[^ ^202^ ^]^ Furthermore, the ZBP1 protein functioned as an endogenous/viral nucleic acid ligand sensor, which modulated innate immune responses.^[^ ^203^ ^]^ Upon its activation, RIPK3 and caspase‐8 were recruited to stimulate NLRP3 inflammasomes, resulting in necrosis and pyroptosis.^[^ ^204^ ^]^ Another study highlighted the impact of elesclomol treatment on CRC cells, which caused an elevation in mitochondrial Cu(II) levels and reduced the expression of Cu(II) transporter ATP7A, promoting ROS buildup. This signaling pathway culminated in the degradation of SLC7A11, ultimately leading to ferritin‐depleted cell death.^[^ ^113^ ^]^ GOx@[Cu (tz)] catalytic activity can only be triggered and consumed in high GSH levels within cancer cells. The ensuing redox process between Cu(II) and intracellular GSH induces GSH depletion and Cu (I)‐mediated Fenton reaction, resulting in the synthesis of hydroxyl radicals from H_2_O_2_.^[^ ^205^ ^]^ As a result, glucose consumption and glutathione depletion lead to ferroptosis in cancer cells.^[^ ^206^ ^]^ These outcomes signified that there indeed existed an intricate set of interactions underlying different forms of PCD.

## 9 Activators and Inhibitors Targeting Ferroptosis, Necroptosis, and Pyroptosis in GI Cancers

**Figure 7:**
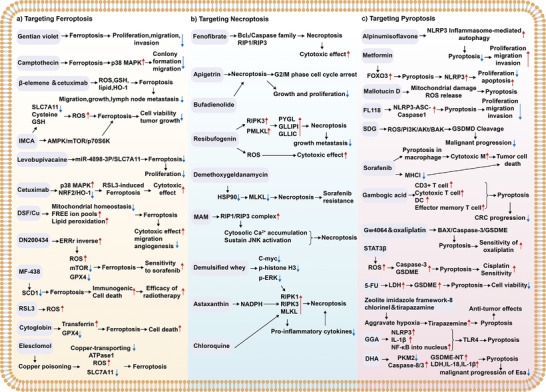
The small molecule compounds or other drugs that play an important role in cancer treatment by targeting ferroptosis, necroptosis and pyroptosis. These small molecule compounds or other drugs mentioned in this review which played an important role in cancer treatment by targeting ferroptosis, necroptosis and pyroptosis are summarized. a) Targeting ferroptosis: Gentian violet, Camptothecin, β‐elemene & cetuximab, IMCA, Levobupivacaine, Cetuximab, DSF/Cu, DN200434, MF‐438, RSL3, Cytoglobin, and Elesclomol. b) Targeting necroptosis: Fenofibrate, Apigetrin, Bufadienolide, Resibufogenin, Demethoxygeldanamycin, MAM, Demulsified whey, Astaxanthin, and Chloroquine. c) Targeting pyroptosis: Alpinumisoflavone, Metformin, Mallotucin D, FL118, SDG, Sorafenib, Gambogic acid, Gw4064 & oxaliplatin, STAT3β, 5‐FU, Zeolite imidazole framework‐8, GGA, and DHA.

The development of novel antitumor drugs that aimed to target ferroptosis, necroptosis, and pyroptosis is a multifaceted process that requires rigorous testing and validation. Recent investigations suggested that many clinically approved medications exhibit potent antitumor effects by inducing or inhibiting inflammatory regulated cell death (RCD) modalities during preclinical studies. Specifically, in CRC cell lines, chloroquine (CQ) has been found to upregulate endogenous receptor‐interacting protein kinase 3 (RIPK3), which promoted necroptosis independent of apoptosis inhibitors.^[^ ^207^ ^]^ Similarly, metformin inhibited cancer cell proliferation by inducing mitochondrial dysfunction and heat‐stimulated cell death via the AMP‐activated protein kinase/sirtuin 1/nuclear factor‐kappa B signal transduction pathway, which activated caspase‐3 and the production of gasdermin E‐pore‐forming domain (GSDME‐PFD).^[^ ^208^ ^]^ Furthermore, Lu Wang et al. demonstrated that metformin targeted the miR‐497/proline‐, glutamic acid‐, and leucine‐rich protein 1 (PELP1) axis to induce focal pyroptosis death in ESCC cells.^[^ ^199^ ^]^ Sorafenib, an approved drug for the treatment of HCC and RCC, acts by inhibiting system Xc‐, leading to iron deficiency anemia by impeding glutathione (GSH) production.^[^ ^209^ ^]^ Sorafenib also cooperates with sulfasalazine, a simulant of amino acid exchange, to prevent the activation of branched chain amino acid aminotransferase, a key enzyme involved in thioamino acid metabolism, thereby inducing deionization of HCC cell lines both in vitro and in vivo.^[^ ^210^ ^]^ Notably, elesclomol, a copper ionophore, has been reported to induce copper poisoning in various types of cancer cells by inducing proteotoxic stress, while promoting Cu‐dependent ferroptosis in CRC cells by inducing degradation of copper transport ATPase 1 and thereafter facilitating ROS accumulation and degradation of SLC7A11, as shown by Gao Wei et al.^[^ ^113^ ^]^ Several small molecule compounds that target necroptotic cell death are undergoing testing, including the butadiene lactone family compound resibufogenin, which significantly inhibits the proliferation of CRC cell lines by upregulating RIPK3 expression.^[^ ^139^ ^]^ Inhibitors of regulated cell death have also been investigated, although only limited studies have been conducted in GI cancer cells. Clinical trials are still in their infancy for novel activators and inhibitors of RCD, despite the recent publication of studies on this topic. For instance, clinical trial no. NCT04229992 aimed to examine the efficacy of a necrosis inducer in CRC, while aspirin, a necrosis inducer, was evaluated in a phase II trial in patients with colorectal adenoma (NCT02965703). Although modulators of these two forms of RCD have been explored in clinical trials, no results have yet been reported. With the advent of high‐quality studies on cell death pathways, more clinical trials will be conducted to understand these four cell death modalities. We believe that a better comprehension of ferroptosis, necroptosis, and pyroptosis could lead to improved anticancer treatment in the near future. These small molecule compounds or other drugs mentioned in this review which played an important role in cancer treatment by targeting the newly discovered RCD modalities were also summarized in **Figure** **7**.

## 10 Conclusions and Prospectives

Ferroptosis has been extensively studied to determine its underlying mechanisms and signaling pathways. Ferroptosis is regulated by iron and lipid metabolism along with various signaling pathways, and it plays a vital role in many pathophysiological processes. For cancer treatment, the induction of ferroptosis in cancer cells is a promising strategy because it inhibits tumor growth and proliferation. Ferroptosis‐targeting drugs are rapidly growing worldwide. These drugs possess potential as a therapeutic intervention for cancer patients and have the potential to become the mainstay of anticancer strategies. Although currently developed ferroptosis‐targeting drugs have issues such as poor bioavailability and limited targeting, developing combinations of small‐molecule compounds with specific receptors for cancer cells will assist in overcoming the limitations. Recently developed therapies have combined ferroptosis‐targeting drugs with conventional immunotherapy or radiation therapy for significant tumor suppression, contributing to the development of conventional cancer therapies. These findings suggest that ferroptosis is a potential target to develop novel anticancer therapies. Necroptosis is a crucial and autonomous host cellular defense mechanism that can effectively inhibit pathogens. Numerous studies have identified the necroptosis pathway and nodal proteins, elucidated the effects of necroptosis on overall organismal physiology, and assessed the inflammatory response initiated by necroptosis. However, to date, the molecular mechanisms that regulate immune cell phenotypes and the relationship between necroptosis and inflammatory responses require further clarification. The interplay between necroptosis and apoptosis adds complexity to the relevant pathways, including the intricate relationship between caspase‐8, FADD protein, and RIPK1 in the apoptosis and necroptosis pathways. The possible regulatory functions of other apoptotic checkpoints, such as caspase‐9, Bal2, and Bax, in programmed necrosis, warrant further investigation. Additionally, given the tissue variability of necroptosis and its association with the development of diverse pathologies, precise treatment may be guided by detailed knowledge of the triggers of necroptosis in different cell types and the underlying mechanisms. Considering the experimental treatment of certain diseases, several inhibitors of the necroptosis pathway have been developed, including purified Chinese herbal medicine preparations and chemical small‐molecule necroptosis activators or inhibitors. Nonetheless, further investigation is necessary to fully elucidate their targets and mechanisms of action.

The treatment of GI cancers involves various modalities such as surgery at early stages and targeted drug therapy at late stages. Targeted drug therapy hinges on key processes such as cell death. Ferroptosis, necroptosis, and pyroptosis are involved in the therapeutic activity of targeted drug therapies for GI cancers. More specific studies on their regulation are warranted before their clinical use. Nevertheless, emerging evidence suggests that inducers of these forms of PCD hold great promise for the treatment of GI cancers. The genes that related to ferritin metabolism can impact the prognosis of GI cancers. Targeted therapies using RNA and nanoparticle technologies are effective in inducing ferroptosis and may provide a novel opportunity for the treatment of GI cancers. Moreover, inducing PCD also seems to be a very promising strategy to overcome chemoresistance and inhibit GI tumor development induced by antiapoptosis.^[^ ^211^ ^]^ However, there is a dark side of inducing nonapoptotic PCD as the novel strategies of GI cancers therapy. The cytokines or DAMPs (high‐mobility group box 1, S100 proteins, and heat shock proteins) released from cancer cells might exacerbate GI tumors progression.^[^ ^212^, ^213^ ^]^ As mentioned above, pyrolytic cells not only function as tumor‐associated antigens by releasing cytokines and proinflammatory factors, but also serve as danger signals, releasing DAMPs that act as important immune adjuvants for the recruitment and maturation of APCs.^[^ ^176^ ^]^ Furthermore, DAMPs may further mediate GI cancers progression by activating chronic inflammation pathways and releasing IL‐1, IL‐6, LT‐β, IFN‐γ, TNF, and transforming growth factor (TGF)‐β.^[^ ^214^, ^215^ ^]^ Therefore, Further research needs to focus on overcoming above issues to improve the therapeutic effects and avoid adverse effects, and using DAMPs “the double‐edged sword” well. Also, we will continue to unravel the regulatory mechanisms of ferroptosis, necroptosis, and pyroptosis, and believe new interventions and strategies that target these processes will be available for the treatment of GI cancers in the near future.

## Conflict of Interest

The authors declare no conflict of interest.

## Author Contributions

Original draft preparation, allocation, revision, supplement and edition: S.L. and X.Z. All authors have read and agreed to the published version of the manuscript.
